# Improving Diabetic Foot Care With Infrared Thermography and Artificial Intelligence: A Review

**DOI:** 10.1177/19322968261432639

**Published:** 2026-04-03

**Authors:** Pedro Teixeira, Vítor Filipe, Ana Teixeira

**Affiliations:** 1 University of Trás-os-Montes e Alto Douro, Vila Real, Portugal; 2 INESC TEC—INESC Technology and Science, Porto, Portugal; 3 Centro de Matemática, Universidade do Minho – Polo CMAT-UTAD, Vila Real, Portugal

**Keywords:** deep learning, diabetic foot, machine learning, neural network, thermography

## Abstract

**Background:**

One of the most common consequences in individuals with diabetes is the diabetic foot, which can cause foot ulcers and even lead to limb amputation. Since an increase of the temperature in the plantar region is directly correlated with an increased risk of ulceration, infrared thermography (IRT) has been used in multiple studies as an automatic tool for detecting problems in diabetic foot. Artificial intelligence-based computer-aided diagnosis systems are being more frequently used to improve decision-making and minimize errors. These technologies are designed to increase examination accuracy, consistency in image interpretation, prognosis evaluation support, and examination accuracy. They also have the ability to offer insightful information and help medical professionals to manage diabetic foot issues successfully.

**Methods:**

In this work, 37 papers that used thermography and artificial intelligence (AI) to identify diabetic foot complications and/or predict the risk of developing diabetic foot are analyzed.

**Results:**

The results demonstrate the potential of IRT imaging implementation with AI for the identification and prediction of diabetic foot complications.

**Conclusions:**

The combination of IRT and AI shows significant potential for diabetic foot assessment; however, the great majority of these studies show that the research is confined to classification of foot thermograms using pre-prepared data sets. In particular, there is limited research on segmentation methods and constraints in the use of deep learning due to the lack of large and diverse datasets.

## Introduction

Diabetes mellitus (DM), also referred to as diabetes, is a chronic condition characterized by the body’s inability to use its main energy source, glucose (sugar), properly, resulting in increased blood glucose levels (hyperglycemia). Diabetes is a chronic disease that can have catastrophic implications if not managed properly.^1^ Individuals with diabetes can be classified according to the type of diabetes they have. The two most common types of diabetes are “insulin-dependent” or type 1 diabetes and “non-insulin-dependent” or type 2 diabetes.

The diabetic foot, which can be characterized as infection, ulceration, and/or destruction of deep tissues, is one of the most common problems observed in individuals with diabetes.^2^ Between 12% and 25% of individuals with diabetes are at risk of developing foot ulcers during their lifetime, which are mostly linked to peripheral neuropathy and frequently to peripheral vascular disease.^3^

Diabetic foot ulcers (DFUs) can be prevented with early identification and treatment. As a result, conducting regular evaluations is critical. However, there are a variety of constraints that may be connected with diabetes-related health problems or any social challenges, making self-examination difficult.^4^ An increase in skin temperature can also indicate tissue damage or inflammation caused by trauma or excessive pressure on the foot. As the risk of ulceration is directly related to increased temperature in the plantar region, infrared thermography (IRT) is one of the non-invasive methods that can be used to predict risk, as temperature differences in the foot can indicate problems associated with diabetic foot.^5^

Artificial intelligence (AI) refers to the utilization of technology and computers to emulate intelligent behavior and critical thought. Different approaches deal with various and expanding volumes of health data, enabling greater patient autonomy and individualized care. The diagnosis and prognosis of DM and its consequences, such as diabetic foot, have been studied.^6,7^ Due to the simplicity of high-volume data collecting and sophisticated computational processing, the automation of health care administration has caused a change both in the industry and the development of AI-based solutions. It made possible to predict delayed diagnoses and locate preventive therapies. These prediction models can be used in clinical practice to better identify the individuals with diabetes at high risk, who need closer monitoring and more intense care.^8^

In this article, a review is presented, with the aim to emphasize the potential of combining AI with IRT imaging for the diagnosis and prediction of diabetic foot problems, while also recognizing its current limitations.

In the work of Gosak et al,^8^ articles that address the prediction of the risk of developing DFU using AI techniques are reviewed, but limited to the studies that use thermography with models just to predict the risk of developing DFU or to classify thermograms. In this review, studies that can be used to identify foot complications, including segmentation and classification techniques, are also included.

This review is structured as follows: The “Materials and Methods” section describes the search strategy, inclusion/exclusion criteria, and data extraction process. The “Results” section presents the findings, beginning with an overview of the target groups studied, followed by a description of how the foot is divided into anatomical regions. It, then, categorizes the analyzed literature based on the techniques applied, including studies focused exclusively on segmentation, those addressing only classification, and those combining segmentation and classification. The “Discussion” section interprets the main findings and analyzes limitations in current research. Finally, the “Conclusions” section summarizes the main ideas and suggests directions for future work.

## Materials and Methods

For this literature review, the “Web of Science” and “Scopus” databases were searched, as they provide broad coverage of peer-reviewed scientific literature in medical imaging, AI, and biomedical engineering. Only articles written in the English were considered, with no restrictions on the publication year. The search was performed using the query *((“* ***machine learning****”) OR (“* ***deep learning****”) OR (“* ***neural network*** *”)) AND “ thermogra*” AND “ ***diabetic foot****”* applied to the **title, abstract**, and **keywords** fields. This database search identified 106 records, including 42 from “Web of science” and 64 from “Scopus,” primarily published as peer-reviewed journal articles.

Studies were included if they (1) used IRT images, (2) focused on diabetic foot assessment or early detection, (3) applied machine-learning (ML), deep-learning (DL), or neural-network-based classification or segmentation techniques, and (4) included a predictive modeling component.

**Figure 1.**
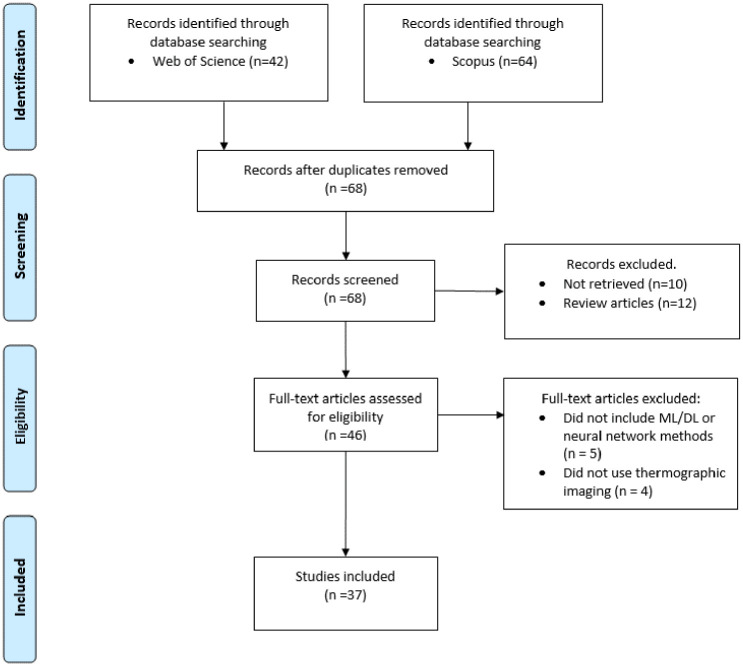
PRISMA flow diagram.

Figure 1 presents a PRISMA (Preferred Reporting Items for Systematic Reviews and Meta-Analyses) flow diagram^9^ summarizing the identification, screening, eligibility, and the inclusion/exclusion stages of the literature search. A total of 106 records resulted from the initial search, with duplicates removed in the first phase, leaving a total of 68 articles for screening. During the screening stage, review articles and records for which the full text was not available were excluded. During the eligibility (full-text assessment) stage, studies were excluded if they did not involve IRT images, did not have a clear focus on early detection, did not implement classification or segmentation techniques via ML, DL, or neural networks, or did not include a predictive modeling component. As a result, a total of 37 articles were included in the final review.

## Results

The following subsections describe the cataloging that was performed; a synthesis of the main topics based on the chosen characteristics is presented.

The articles were categorized into three main groups, accordingly to the used techniques: only segmentation techniques, only techniques of classification and both techniques combined.

### Target Group

Depending on their specific research objectives, the included studies were categorized based on the population under analysis: some focused exclusively on individuals with diabetes, while others focused on individuals without diabetes or healthy individuals, while several included both groups:

- Studies that aim to investigate specific aspects related to diabetic foot conditions, such as temperature variation with different types of complications in individuals with diabetes,^10^ focus exclusively on this population.
- Some studies included individuals without diabetes or healthy individuals as a comparison group to enable the analysis of thermographic differences, particularly in foot temperature distribution, between populations with and without diabetes. In such cases, the primary objective was to identify specific thermal patterns or anomalies associated with diabetic conditions by contrasting them with those observed in healthy individuals, especially in the context of foot complications.

### Foot Division Into Regions

Several studies have conducted thermal analysis based on the observation of specific points or specific foot regions. Considering the temperature of the entire foot allows a comprehensive analysis of its overall state. However, since the foot does not have a uniform temperature, it is important to consider regional divisions of the foot.^11^ For example, in studies like^12^ temperatures were recorded in some regions of interest (ROIs) on both feet (focusing on areas with a higher likelihood of ulceration) prior to analysis. However, as one of the main causes of diabetic foot ulceration is the decrease in blood supply, the most commonly used and discussed division, divides the feet into four regions based on the concept of angiosomes, which are tissue regions supplied by a single artery.^13^

### Techniques

An automatic system can easily be connected and processed using various ML and image processing algorithms, such as image segmentation and classification.^14^ These systems involve multiple stages of processing, either in a serial or parallel sequence, for extracting and classifying features from thermal images, enabling health care professionals to access more precise diagnoses.

#### Segmentation

Image segmentation involves dividing an image into several parts/segments with similar characteristics or attributes. This technique forms the basis for image analysis and is an essential step for feature extraction and recognition. Image segmentation techniques can play a crucial role in segmenting and extracting the hottest or coldest regions from clinical infrared images.^15^ For example, the shape, size, and boundaries of the hottest regions in thermal images can help determine the features used to detect abnormalities. So far, various techniques have been applied to extract different regions, such as contouring, background isolation, even the hottest regions in thermograms, and convolutional neural networks (CNNs). By observing the hottest or coldest regions, potentially suspicious regions in real-time thermal images can be of interest to physicians.^15,16^ Another crucial aspect of segmentation is the distinction between a plantar foot and its bottom. It can accurately detect the edges of the foot in thermal images with varying contrast efficiently, laying the foundations for further exploration of the detection or classification of diabetic foot status.^17^ Table 1 summarizes the studies that utilize segmentation techniques.

**Table 1.** Basic Characteristics of the Included Studies that Use Segmentation Techniques.

	Study aim	Techniques	Data set	Main contributions	Limitations/ future work
Cao et al^17^	To develop and evaluate new segmentation methods for cold-stressed active thermography in diabetic foot diagnosis using deep neural networks.	Plantar foot segmentation network (PFSNet)	1800 plantar foot thermal images	A novel PFSNet for thermal image segmentation, combining U-shaped networks, multi-scale feature extraction, and attention modules, outperformed existing methods, achieving 97.3% IOU and 95.4% DSC without complementary data.	The study aims to expand recruitment due to its small sample size and complex cold immersion procedures, and requires further research on scaling and clinical implementation.
Maheswari and Kayalvizhi^16^	To develop a robust and automated segmentation model for diabetic foot ulcers using a deep neural network based on U-Nets and modified Swin Transformers. The goal is to accurately segment ulcer-prone areas from thermal images.	U-Net Swin transformer models	INAOE data set (public): 167 individuals: 122 with diabetes and 45 with non-diabetes	The study developed a U-Net and modified Swin Transformer-based architecture for thermal image segmentation, improving accuracy through feature extraction and integration. Achieved metrics: 99.5%, IoU: 98.9%, precision: 99.33%, and recall: 99.56% for diabetic thermal ulcer image segmentation.	The study suggests further exploration of lightweight transformers, improving structural pixel-level feature learning, testing on diverse data sets, and optimizing the model for real-world clinical scenarios.
Maldonado et al^15^	To develop a non-invasive monitoring system for diabetic foot that can detect and classify temperature differences in foot sole zones as ulcerous or necrotic, without the need for a controlled environment or homogeneous background.	Mask R-CNN	Two data sets: 108 images obtained from 17 individuals with and without diabetes. 141 images from 47 individuals with and without diabetes.	The study successfully detects and visualizes risk zones in diabetic foot patients, identifying ulcers and necrosis in foot sole zones, with 90% accuracy for ulcers and 88% for necrosis, demonstrating the feasibility of deep-learning algorithms for segmentation.	The system’s effectiveness may be limited by its reliance on pre-trained models, and future work aims to adapt the algorithm for smartphone-based remote monitoring and diagnosis.
Arteaga-Marrero et al^18^	To evaluate segmentation approaches for diabetic foot disorders using multimodal images (visual-light, infrared, and depth) and overcome technical challenges related to foot sole segmentation.	U-Net, Skin, U-Net + Depth (UPD), Skin + Depth (SPD)	74 images from 37 healthy individuals.	The U-Net and Skin segmentation approaches, optimized for spatial information, showed satisfactory performance, while the SPD approach showed comparable or superior results, making the UPD approach preferred for replacing manual feet segmentation.	The study does not consider partial foot amputations or deformations, but they may cause morphological and functional differences in temperature patterns analysis, requiring further investigation.

#### Classification

In this context the term “classification” is used to describe the procedure of grouping patients’ plantar thermograms according to their state of health. The majority of articles specifically seek to categorize the thermograms as belonging to both healthy individuals and individuals with diabetes.

To perform this categorization, both ML and DL models have been applied to plantar thermogram analysis. Support vector machines (SVMs) classifiers have been widely employed for the binary classification of healthy and diabetic feet,^12,19^ whereas K-nearest neighbors (KNNs) and artificial neural networks (ANNs) classifiers have also been employed for the classification of diabetic feet based on thermal patterns.^20,21^ Ensemble methods like AdaBoost and Random Forest classifiers are another approach investigated for enhancing the robustness and accuracy of diabetic foot screening systems.^22,23^

More recent studies have been using DL methods, especially CNNs, because of their success in the classification of thermographic images. Some studies have utilized transfer learning using established CNN models, including AlexNet,^24^ VGG networks,^25^ ResNet models,^26^ DenseNet,^27^ and MobileNet models.^28^ Moreover, more advanced models like DarkNet-based networks^29^ and vision transformers (ViT) have recently been explored for the classification of plantar thermograms, and they have shown promising results on small data sets.^30^ During the training process, the classification model extracts distinctive thermal features from the labeled plantar thermograms, which are, then, utilized for distinguishing between the healthy and diabetic classes or between the stages of diabetic foot complications. After the training process, the model can be used for predicting the health status of patients from unseen thermograms. Table 2 summarizes the studies that utilize classification techniques.

**Table 2.** Basic Characteristics of the Included Studies That Use Classification Techniques.

	Study aim	Techniques	Database	Main contributions	Limitations/future work
Carlos Padierna et al^12^	To propose a non-invasive methodology for the characterization of peripheral arterial disease (PAD) in patients with type 2 DM using IRT and ML techniques	SVM	23 individuals with diabetes type II	Achievement of an average accuracy of 92.64%, sensitivity of 91.80%, and specificity of 93.59% in PAD classification. Availability of experimental data and source code for easy implementation as a supporting tool for physicians in PAD identification.	Future work consideration of combining PAD assessment with diabetic neuropathy
Prabhu and Verma^31^	To propose a deep-learning framework for automatic classification of healthy skin versus DFU using plantar thermogram.	DenseNet VGGNet MatConvNet	INAOE data set (public): 167 individuals: 122 with diabetes and 45 with non-diabetes	A deep-learning model for DFU classification using plantar thermograms achieved 97.9% accuracy, comparable or superior to existing algorithms like DenseNet, VGGNet, and MatConvNet. This automated system shows potential for clinicians in foot screening and quick preventive actions.	The system’s performance may be impacted by complex conditions, and future work should focus on optimizing the model for various environments and enhancing embedded device deployment.
Khandakar et al^28^	To develop a machine-learning-based framework for early detection of DFU using thermogram images	MobilenetV2 AdaBoost Classifier	INAOE data set (public): 167 individuals: 122 with diabetes and 45 with non-diabetes	The study compares machine-learning-based scoring techniques with feature selection and optimization techniques and learning classifiers. It proposes a robust solution for identifying diabetic feet using MobilenetV2 CNN and AdaBoost Classifier, achieving high *F*1-scores.	The system’s effectiveness is limited to clinical trials, with future work focusing on optimizing thermogram features and expanding smartphone deployment.
Khandakar et al^25^	To develop a machine-learning-based approach for classifying thermogram images of diabetic foot based on severity of foot complications, with the aim of early detection and reliable stratification.	VGG 19 CNN	INAOE data set (public): 167 individuals: 122 with diabetes and 45 with non-diabetes	The study compared classical ML algorithms with feature engineering and CNN with image-enhancement techniques. The VGG 19 CNN model achieved high accuracy, precision, sensitivity, *F*1-score, and specificity of 95.08%, 95.08%, 95.09%, 95.08%, and 97.2%, respectively, in stratifying diabetic foot complications, while the proposed stacking classifier showed comparable performance.	Future research aims to deploy the system as a web application for remote health care.
Jain and Sreedevi^24^	To analyze the performance of transfer learning deep-learning networks and propose an enhanced methodology (ProNet) for classifying diabetic foot thermograms.	AlexNet, ResNet-101, ProNet,	INAOE data set (public): 167 individuals: 122 with diabetes and 45 with non-diabetes	The ProNet methodology, a combination of AlexNet and ResNet features, achieved a higher accuracy of 98.9%, outperforming previous methods like ResNet-101, enhancing precision, specificity, and *F*1-score.	Future suggestions include improving the DL algorithm structure, acquiring more foot thermograms, investigating DFU classification, classifying foot images based on thermal change index (TCI) values, and using a portable IR camera.
Jain and Sreedevi^29^	To evaluate the performance of transfer learning deep-learning networks (DarkNet-19 and DarkNet-53) and propose an improved methodology (Pro-Multi-Net) for multiclass classification of diabetic foot thermograms based on (TCIs)	DarkNet-19, DarkNet-53, Pro-Multi-Net	INAOE data set (public): 167 individuals: 122 with diabetes and 45 with non-diabetes	The Pro-Multi-Net algorithm, combining DarkNet-19 and DarkNet-53 features, achieved higher accuracy and performance metrics than existing DL models. DarkNet-53 demonstrated residual layer effectiveness in degrading gradient problems, achieving 95.5% overall accuracy.	Future scope suggestions for enhancing the network architecture, classifying thermograms with distinctive color and texture features, tuning parameters, and collecting a greater number of images.
Filipe et al^20^	To develop a functional methodology for the analysis and classification of different thermal changes in the plantar region of individuals with diabetes and healthy individuals.	Support SVM, KNN	INAOE data set (public): 167 individuals: 122 with diabetes and 45 with non-diabetes	The two proposed models performed well, but compared with model 1 (thermogram), model 2 outperforms model 1 as it allows a better classification of healthy individuals and individuals with diabetes into the first class. The SVM algorithms performed second best with similar results, followed by the Weighted KNN algorithm; however, this was better than the 3-NN algorithm.	The authors cite as a limitation that the data obtained from the public data set were unbalanced, resulting in under-representation of some classes.
Balasenthilkumaran et al^22^	To develop and compare machine-learning-based algorithms for the diagnosis of diabetes and detection of ulcer-prone regions in the feet using thermal images, and to evaluate their performance.	ANN, linear discriminant, logistic regression, Gaussian naive Bayes.	INAOE data set (public): 167 individuals: 122 with diabetes and 45 with non-diabetes	The study developed machine-learning and image-processing algorithms for diabetes diagnosis and ulcer detection using thermal images. The ANN-based classifier achieved the highest accuracy (93.3%) and *F*1-score (0.95) for diabetes diagnosis.	Future work includes expanding the data set and integrating the algorithms into an application for practical usage.
Anaya-Isaza et al^26^	To explore different strategies for detecting patients with DM using foot thermography.	ResNet50v2	INAOE data set (public): 167 individuals: 122 with diabetes and 45 with non-diabetes	The study uses a new discrimination coefficient based on average temperature, age, and foot temperature to stratify diabetic foot patients. Deep CNNs using ResNet50v2 architecture improve accuracy by up to 17%, emphasizing the importance of data augmentation and transfer learning for foot thermography detection.	The system’s performance relies on data augmentation and transfer learning, with future work focusing on enhancing the thermographic image database for better classification accuracy.
Anaya-Isaza and Zequera-Diaz^32^	To propose a novel method based on modifying the amplitude in the Fourier Transform for data augmentation in deep-learning models for classifying diabetic foot thermograph images.	ANN, DFTNet, ResNet50v2	INAOE data set (public): 167 individuals: 122 with diabetes and 45 with non-diabetes	Introduction of a novel data augmentation method using Fourier transform for deep-learning models. Improved classification performance on diabetic foot thermograph images using the proposed data augmentation. Potential for better classification on limited data sets in thermal pattern classification problems.	The small data set limits atypical subjects’ findings, necessitating validation with a test group to ensure 100% performance or no significant reduction in performance.
Muralidhara et al^33^	To present a novel, holistic classification approach that considers thermograms of individuals with and without diabetes based on a CNN.	CNN’s	INAOE data set (public): 167 individuals: 122 with diabetes and 45 with non-diabetes	A comprehensive multiclass classification of thermal imaging of the feet for the prediction and classification of patients with diabetes mellitus were presented. The model achieved the best performance with an overall accuracy of 0.9827, a baseline sensitivity of 0.9684, and a baseline specificity of 0.9892.	The authors present a limitation due to publicly available data, which is often unbalanced and leads to over-represented classes and low sensitivity to under-represented classes.
Munadi et al^34^	The objective of this study is to develop a deep-learning framework for early detection of DFU using thermal images and decision fusion.	MobileNetV2, ShuffleNet	INAOE data set (public): 167 individuals: 122 with diabetes and 45 with non-diabetes	The proposed framework achieves 100% accuracy in classifying DFU thermal images using a decision fusion method that combines MobileNetV2 and ShuffleNet classification results, enhancing accuracy by approximately 3.4% compared to baseline models.	Future research prospects include enriching the data set for improved generalization and exploring additional fusion strategies for smaller CNN models suitable for mobile devices.
Khandakar et al^27^	To explore machine-learning approaches for classifying thermogram images of diabetic foot based on the TCI, with the aim of early diagnosis and severity classification of diabetic foot complications.	SVM, KNN, Resnet18, ResNet50, DenseNet201	INAOE data set (public): 167 individuals: 122 with diabetes and 45 with non-diabetes	The proposed machine learning framework outperformed a state-of-the-art DFTNet in classifying thermogram images, with a 90.1% accuracy rate, surpassing literature-reported performance. Classical ML models also demonstrated exceptional performance compared with CNN models without image enhancement.	The system has the potential for remote health care monitoring of individuals with diabetes from their homes but validation with new data sets and different infrared cameras is recommended for further robustness.
Christy Evangeline et al^35^	To evaluate the feasibility of using non-contact thermography as a screening modality for diabetic foot syndrome (DFS) and develop an intelligent system for automatic classification of thermal patterns in subjects with diabetes	RBF Net	153 individuals with diabetes types II and 38 without diabetes.	An intelligent system was developed for automatic thermal pattern classification in individuals with diabetes, achieving cross-validation accuracies of 98.89%, 95.2%, and 97.1% for various tasks, demonstrating its effectiveness in accurately classifying thermal patterns and its potential for mass screening.	The system’s performance relies on data augmentation to address data imbalance, with future work focusing on enhancing classification models and refining thermal distribution analysis for improved early diagnosis.
Alshayeji et al^36^	To develop an accurate, fast, and reliable end-to-end traditional ML model for real-time CAD applications of DFU using thermal images.	SVM	INAOE data set (public): 167 individuals: 122 with diabetes and 45 with non-diabetes	Development of an end-to-end ML model for early detection of DFU using thermal images. The proposed model is able to detect minute temperature variations across the foot and identify DM patients susceptible to DFU during the early stages. Achievement of high accuracy and speed for real-time DFU detection.	The system’s effectiveness relies on various invariant feature techniques and data augmentation, with future work focusing on enhancing early diagnosis and integrating the CAD system into online solutions.
Hernandez-Guedes et al^37^	To extract relevant features from infrared thermograms for the classification of DFUs and evaluate their performance compared with state-of-the-art features	SVM	INAOE data set (public): 167 individuals: 122 with diabetes and 45 with non-diabetes	The study used a SVM classifier to classify subjects at risk of ulcers, with concrete drop-out showing the best feature extraction performance, achieving an F1 score of 90%, with slightly better performance than previous reports.	The study’s findings are promising but limited by data set specificity, suggesting future research should validate feature extraction methods across diverse populations to enhance clinical applicability.
Khosa et al^23^	To recognize diabetic foot ulcers using publicly available thermographic image data with machine-learning and deep-learning approaches.	SVM, Random Forest, XGBoost, Naive Bayes, AdaBoost, KNN, ResNet50, DenseNet121	INAOE data set (public): 167 individuals: 122 with diabetes and 45 with non-diabetes	SVM classifier outperformed other machine-learning techniques, and machine-learning models achieved higher accuracy at the full-image level. Among deep-learning models, the proposed CNN-based model demonstrated the best results in terms of area under the curve (AUC) and accuracy.	The study acknowledges the limitations of thermogram images without pre-processing and suggests that while the machine-learning model can provide a reliable second opinion, it cannot replace human expertise.
Shao^30^	To enhance the early detection of diabetic foot using thermograms by improving the performance of the original vision transformer through the addition of a learnable block.	Vision transformer	INAOE data set (public): 167 individuals: 122 with diabetes and 45 with non-diabetes.	The proposed ViT achieved an accuracy of 99%, outperforming other ViTs and approaching the level of CNNs. Results indicated that the original ViT struggled with small-size data sets, and the proposed ViT with an added block significantly enhanced performance.	Future research aims to further improve ViTs’ inductive ability and test on additional small-size data sets.
Reyes-Luévano et al^38^	To propose a novel CNN architecture, DFU_VIRNet, for automatic classification of abnormal skin (DFU) versus normal skin (healthy skin) using visible and infrared (thermography) images. In addition, to introduce a method based on estimation maps for detecting risk zones with a high probability of the patient developing DFU.	DFU_VIRNet	3745 samples for the visible data set and 4800 samples for the infrared data set. It contains images of healthy feet and two DFU diseases, ischemia and infection.	DFU_VIRNet outperformed the current state-of-the-art results with high AUC and F1-score for DFU, ischemia, and Infection classification. The proposed learning mechanism contributed to the high performance.	The study’s findings highlight DFU_VIRNet’s strong performance metrics, but future research should validate its real-world application and enhance accuracy for broader diabetic foot diagnosis.
Castillo-Morquecho et al^19^	To analyze thermal patterns of the foot sole in type 2 diabetes patients using thermography and develop a machine-learning model for classification.	SVM	23 individuals with diabetes types II and 27 without diabetes.	Found correlations between foot temperature, HbA1c, and BMI. Developed a PCA-SVM model with 90% accuracy, 100% specificity, and moderate sensitivity (78.3%). Showcased the potential of thermography and machine learning for diabetic foot assessment.	Small sample size. Future research should include additional measures (eg, ankle-brachial index and physical activity levels) to enhance model accuracy and understanding of temperature mechanisms.
Wei et al^39^	To evaluate and improve the performance of the AlexNet deep-learning algorithm in classifying diabetic foot ulcers (DFUs) using thermograms.	AlexNet	INAOE data set (public): 167 individuals: 122 with diabetes and 45 with non-diabetes.	AlexNet achieved 97.8% accuracy in detecting DFU on plantar thermograms, demonstrating data augmentation, color vs. greyscale comparison, and hyperparameter manipulation for improved classification accuracy.	The small data set necessitates future work on enhancing classification performance by modifying the deep-learning architecture and increasing the thermogram data set size.
Hernandez-Contreras et al^21^	To propose a novel approach to characterize temperature patterns in thermographic foot images, supporting the early diagnosis and follow-up of individuals with diabetes.	ANN	44 thermograms (24 participants without diabetes and 20 participants with diabetes).	The study developed feature vectors using 3D morphological pattern spectrum and relative position to classify groups with and without diabetes, achieved a classification rate of 94.33% using a neural network with Levenberg-Marquardt backpropagation algorithm, and demonstrated a low computational cost approach.	The database is expanding to include more healthy individuals and individuals with diabetes at various stages, with future directions including improved classification accuracy and feature vector refinement.
Katual and Kaul^40^	To develop and evaluate a parallel CNN for the classification of diabetic foot thermograms, comparing its performance with AlexNet and GoogleNet.	Parallel CNN AlexNet GoogleNet	INAOE data set (public): 167 individuals: 122 with diabetes and 45 with non-diabetes.	A parallel CNN with three convolutional layers achieved an accuracy of 91.04%, outperforming standard AlexNet and GoogleNet. Max-pooling layers reduced dimensionality and improved accuracy. Performance metrics were analyzed for effectiveness.	The proposed model has slightly longer training time than AlexNet and could benefit from further improvements in parallel layer number and data set expansion for more robust results.
Aferhane et al^41^	To apply CNN-based affine registration methods for aligning thermal images of the plantar foot to detect early signs of diabetic foot ulceration. The study evaluates both contralateral and multitemporal foot registration methods.	Affine ConvNet VGG-16 AIRNet DenseNet121	102 healthy individuals 145 individuals with diabetes.	Achieved 95% Dice Similarity Coefficient for contralateral and multitemporal registration, demonstrated robustness of CNN models, and conducted clinical study classifying patients into ischemic and non-ischemic groups based on thermal image analysis.	The study suggests future work to enhance multitemporal registration performance by expanding the data set and integrating registration models with segmentation tasks.
Christy Evangeline and Srinivasan^42^	To compare off-the-shelf networks and a custom DPN-Net for classifying healthy versus neuropathic feet using thermal images.	DPN-Net MobileNet ResNet-50	153 individuals with diabetes	DPN-Net, a custom network, achieved 98.5% test and validation accuracy, outperforming MobileNet and ResNet-50 in classification accuracy and computational efficiency, demonstrating its potential for telemedicine.	Future research could enhance DPN-Net’s telemedicine mobile application, classify neuropathy severity, detect thermal asymmetry, and investigate auto-detection of ulcer hotspots.
Hernandez-Guedes et al^43^	To propose a method for evaluating deep-learning models’ performance on small, scarce data sets and detect overfitting beyond traditional metrics.	Deep neural network (DNNs)	INAOE data set (public): 167 individuals: 122 with diabetes and 45 with non-diabetes IATEC data set: 74 images from 37 healthy individuals.	The IB approach was used to evaluate models’ generalization in small data sets, revealing that only two out of five models consistently performed, and skip-connections significantly impacted model performance in diabetic foot ulcer samples.	The evaluation framework will be expanded to new data sets with similar scarcity constraints, and models (PCAE and PFCAE) will be scaled with larger data sets.
Sharma et al^44^	To develop and test a novel deep-learning model Plantar Foot Ulcer Thermogram Network Architecture (PFUTnet) for assessing the severity of diabetic foot ulcers (DFUs) using thermal imaging.	PFUTnet Inception V3 AlexNet VGG-16, VGG-19	71 individuals with diabetes and 33 individuals without diabetes.	PFUTnet, a novel deep-learning architecture, outperformed Inception V3 and AlexNet with an AUC score of 0.98. It used parallel convolutional layers for better pattern capture and developed a new thermal data set for DFU detection in Indian health care settings.	The study was tested on a small sample of 104 subjects, and further validation is needed on a larger data set and in more clinical settings.
Fasihi-Shirehjini and Babapour-Mofrad^45^	To evaluate the efficiency of ConvNeXt variants for automatically detecting diabetic feet from thermal images, and to develop a CAD system for early diagnosis of diabetic foot ulcers.	ConvNeXts	INAOE data set (public): 167 individuals: 122 with diabetes and 45 with non-diabetes.	The study introduced ConvNeXt variants for feature extraction from thermal images of diabetic and healthy feet. It explored logistic regression, support vector machine, and fully connected layers as classifiers. Fancy PCA was proposed as a data augmentation technique, achieving 100% accuracy when paired with ConvNeXt XLarge and LR classifier. The model demonstrated high classification accuracy.	The CAD system’s small, unbalanced data set may introduce bias in predictions, suggesting further exploration of other feature extractors and classifiers. It should be considered an auxiliary tool for clinicians, not a substitute for medical evaluation.

## Segmentation and classification

Some studies integrate both segmentation techniques and classifications to present more complete systems. In these methods, segmentation is employed as a pre-processing step to detect ROIs, as explained in the “Segmentation” section, and classification is, then, carried out using the information obtained from these regions.

Unlike the methods explained in the “Classification” section, where classification is carried out directly on the whole thermogram or on pre-defined ROIs for the plantar area, combined segmentation and classification methods are used when there is a need to detect localized thermal irregularities before making any decisions. In these scenarios, segmentation helps in the classification process by demarcating ROIs that are of anatomical or thermal significance, while classification offers subject-level or region-level diagnostic results.

In diabetic foot analysis, segmentation is particularly beneficial when the aim is to examine localized patterns, such as asymmetric angiosomes, ulcerative prone areas, or localized areas of temperature increase. On the contrary, classification only methods are adequate for global screening applications, such as distinguishing normal subjects from ones with diabetes without the need for spatial localization.

Some methods combine both segmentation and classification by first segmenting to define ROIs, followed by classification using ML or DL models, thereby offering both spatial localization of irregular regions and good classification accuracy.^14,46-49^ Table 3 summarizes the studies that utilize segmentation and classification techniques in combination.

**Table 3.** Basic Characteristics of the Included Studies That Use Segmentation and Classification Techniques.

	Study aim	Techniques	Data set	Main contributions	Limitations/future work
Cruz-Vega et al^46^	To compare the performance of SVM, multilayer perceptron (MLP), and DL structures in the classification of thermal images of diabetic foot.	Segmentation: - Evolutionary algorithms Classification: - SVM - MLP - AlexNet	No mention	The study compares SVM, MLP, and DL in binary thermal image classification, finding satisfactory accuracy. The automatic segmentation procedure provides well-defined ROIs for diabetic foot patients and the control group. SVM and MLP achieve high accuracy after feature extraction and segmentation. DL structures outperform in terms of classification accuracy and eliminate the need for ROI definition and feature extraction.	While DL structures show improved accuracy and reduced training time, future work should address challenges in extracting relevant regions in thermal images for better automatic classification.
Nag et al^47^	To develop an approach for early detection of diabetic foot ulcers using infrared thermography and machine-learning techniques	Segmentation: - Lazy snapping Classification: - SVM - K-NN - Decision tree (DT)	INAOE data set (public): 167 individuals: 122 with diabetes and 45 with non-diabetes	The study used lazy snapping to segment abnormal foot thermograms, evaluating classifiers like SVM, k-NN, and DT. Results showed 97.778% accuracy in normal and diabetic feet, with First Order Statistical features being most effective.	The study shows lower accuracy with GLCM features and 5-fold testing, suggesting future work should improve textural features and validate with diverse data sets.
Cruz-Vega et al^14^	To analyze the use of AI and DL for the classification of diabetic foot thermograms and to analyze the advantages and limitations of this method.	Segmentation: - Histogram-based segmentation Classification: - GoogLeNet - AlexNet - DFTNet	110 thermograms of patients with diabetes.	The DL method outperformed other models and saved time, while CNN methods like GoogLeNet and AlexNet were unsatisfactory. The proposed DFTNet offers satisfactory results in sensitivity, specificity, accuracy, and AUC values.	The authors state that they aim to obtain more images of the thermograms in future studies.
Evangeline et al^48^	To develop an artificial intelligence model that can discriminate between symmetric and asymmetric angiosomes in the plantar feet of subjects with type 2 diabetes using infrared thermograms, with a focus on identifying regions of asymmetry and hotspots indicative of the onset of diabetic foot ulcers.	Segmentation: - Canny edge detection Classification: - SVM - K-NN - Gaussian Naive Bayes - Random forest - Logistic regression	153 patients with diabetes type II	An AI model has been developed to analyze thermal foot images for angiosome-wise analysis, distinguishing between symmetric and asymmetric regions. The model identified hotspots within angiosomes as potential indicators of diabetic foot ulcer onset. The model achieved 98% accuracy, 96.07% test accuracy, and 0.96 *F*1-score.	The model’s effectiveness in detecting asymmetric angiosomes shows promise, but future work should focus on refining region-specific asymmetry prediction and validating the approach across diverse data sets for broader clinical application.
Shan et al^49^	To develop an early screening method for diabetic foot using infrared thermography and improve the accuracy of classification between normal foot and diabetic foot	Segmentation: - Expectation maximization clustering Classification: - ConvNeXt	INAOE data set (public): 167 individuals: 122 with diabetes and 45 with non-diabetes Experimental group of 88 patients with diabetes type II and 45 with non-diabetes	A feature fusion-based early screening method for diabetic foot was developed, enhancing foot features of different scales. The method achieved an accuracy of 97.66% on the experimental data set and 97.01% on the public data set, with the ConvNeXt network outperforming existing networks.	The authors note that in future investigations, they will use the doctor’s diagnosis to make a more detailed division of individuals with diabetes according to the type of complications.

## Discussion

Individuals with diabetes who develop DFUs face significant costs and disabilities. It is crucial for all patients with diabetes to receive comprehensive education about foot care and preventive measures. However, the conventional method of diagnosing DFUs by clinicians and DFU experts is costly and time-consuming. Deep learning in medical imaging offers the potential for automatic DFU diagnosis. Given the complexity of DFUs, AI approaches are well-suited to address issues, such as prompt screening to identify the likelihood of foot ulcers or amputation using appropriate sensor technology.

**Figure 2.**
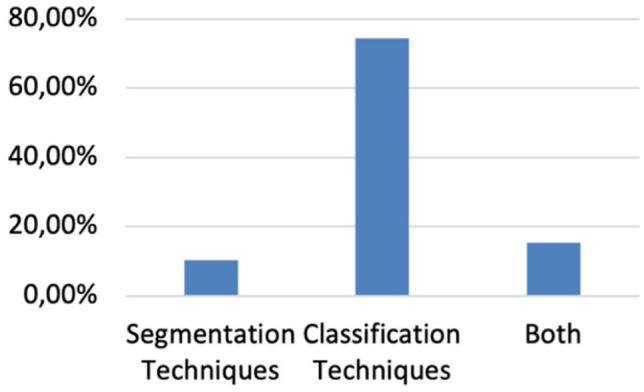
Distribution of the articles according to the type of techniques used.

In this review, articles are categorized as: articles presenting segmentation techniques, articles presenting classification techniques, and articles presenting the combination of both techniques. From Figure 2, it can be observed that the majority of the studies primarily focus on the classification process, with a lack of studies presenting methods for segmenting thermograms with relevant information, such as extracting the foot from the background or isolating the specific foot area required for analysis. The majority of the existing studies focus primarily on classification approaches, while significantly fewer explore segmentation, a fundamental step that can greatly enhance the precision and interpretability of computer-aided diagnostic systems.

In the four studies presented in Table 1, foot images were segmented and analyzed to identify anomalies or areas of interest. Accurate segmentation and localization of foot sole areas were achieved using a variety of techniques, including clustering, Mask R-CNN, U-Net, Skin approaches. Future work for these studies includes further analysis, technique validation, and optimization, with a focus on improving segmentation algorithms, exploring new imaging modalities or approaches, and conducting larger-scale studies to confirm the efficacy and generalizability of the detection techniques.

The studies summarized in Table 2 demonstrate the effectiveness of various ML algorithms, including SVM, CNNs, such as VGGNet, DenseNet, ResNet, and MobileNet, as well as conventional ML methods like AdaBoost classifier, KNN, and logistic regression. These algorithms accurately classify and identify diabetic foot complications based on thermal images of individuals with and without diabetes.

Several studies utilized the public INAOE data set,^11^ which consists of thermal pictures from 167 individuals, including both individuals with and without diabetes. The proposed approaches and algorithms were developed and evaluated using this data set, yielding high accuracies, sensitivities, and specificities ranging from 90% to 100% accuracy in categorizing and stratifying the severity of diabetic foot problems.

However, the studies also acknowledge certain limitations and areas for further investigation. These include the need for larger and more evenly balanced data sets, as well as consideration of under-represented classes to increase sensitivity. The robustness and generalizability of the suggested methods need to be further explored by evaluating them using different infrared cameras, acquisition protocols, and independent data sets. Future research can also explore the integration of other modalities, such as color and texture cues, to improve classification performance and provide a more comprehensive analysis of diabetic foot problems.

Studies that utilize both segmentation and classification techniques in combination highlight the essential role of segmentation in identifying and isolating areas of interest in thermal images. When comparing the performance of various algorithms, it is evident that DL frameworks, such as AlexNet, GoogleLeNet, and the proposed DFTNet, generally outperform more conventional ML techniques, such as SVM and MLP, in terms of classification accuracy. These DL models often eliminate the need for explicit ROI definition and feature extraction, making the classification process more efficient and faster.

However, the use of DL models in diabetic foot diagnosis is still limited by the lack of available data, the cost of data acquisition and annotation, and the need for intensive computational resources.^25,30,43^ These factors partly explain why the use of DL models is less common in this area and the need for continued efforts in data sharing, standardization of data acquisition, and large-scale validation.^27,30^

Moreover, the conditions of image acquisition, such as camera placement, distance, and environment, may cause inconsistencies in thermal distribution.^19,26^ From a clinical standpoint, the implementation of AI-assisted diagnostic systems in a busy clinical setting also poses challenges with regard to workflow compatibility and understanding of the diagnostic output.^35,42^

### Application-Oriented Use Cases of AI Models in Diabetic Foot Thermography

Aside from performance aspects, it can be seen from the reviewed studies that various AI models are appropriate for different application scenarios in the case of diabetic foot thermography.

Lightweight architectures like MobileNet and ShuffleNet are appropriate for resource-limited settings, such as portable devices, smartphone-based screening, and telemedicine applications, because of their low computational complexity and efficient inference.^28,34,42^ These architectures are highly relevant in the context of large-scale screening and telemedicine.

Conversely, more complex architectures of CNNs, such as VGGNet, ResNet, and DenseNet, are more often used in clinical workstation or hospital environments where computational power is less of a constraint.^23-27,31^ These architectures are usually employed in decision support systems for diagnosis.

When the application involves spatial localization and interpretability, such as marking areas that are prone to ulcers or abnormal plantar areas to help with clinical decision-making, it is particularly useful to have frameworks that integrate segmentation and classification.^14,46-49^ In such instances, segmentation offers complementary information to classification.

More contemporary architectures, such as ViT and ConvNeXt-based models, have until now been largely investigated in research environments,^30,45^ and their adoption in clinical practice awaits more data and studies.

For health care professionals, the classification results can serve as a valuable resource for identifying diabetes patients who may be at risk of developing foot issues. Early identification of potential problems enables the implementation of effective preventive measures and interventions to reduce risks and enhance patient outcomes.

It is crucial to remember that the quality and representativeness of the training data, the selection of the ML algorithm, and the choice of classification features all play a role in the effectiveness of the classification process. To ensure the accuracy and generalizability of the model, it should undergo routine validation and continuous improvement.

## Conclusions

Infrared thermography imaging and AI have demonstrated great potential in detecting diabetic foot problems and estimating the potential of developing diabetic foot. The reviewed studies show that AI-based computer-aided diagnosis systems using thermography can help with quick screening and diagnosis of DFUs, giving health care professionals helpful data.

Although the classification process is the main focus of the majority of the papers evaluated, there is a noteworthy dearth of studies on segmentation methods for obtaining pertinent data from thermograms. To create precise computer-aided diagnostic systems, segmentation is a crucial component, and further study is required to enhance this area.

The DL methods are less frequently used in classification research because of the lack of data and the difficulties in acquiring it. The ML techniques like SVM and KNN were frequently used. To effectively use DL approaches, future research should concentrate on gathering larger and more varied data sets.

Health care professionals can use the categorization outcomes from these AI systems as important resources to find individuals with diabetes who are at risk of developing foot issues. Early detection enables the implementation of therapies and preventive measures, improving patient outcomes and lowering the dangers connected with DFUs.
